# Machine-Learning Based Determination of Gait Events from Foot-Mounted Inertial Units

**DOI:** 10.3390/s21030839

**Published:** 2021-01-27

**Authors:** Matteo Zago, Marco Tarabini, Martina Delfino Spiga, Cristina Ferrario, Filippo Bertozzi, Chiarella Sforza, Manuela Galli

**Affiliations:** 1 Dipartimento di Elettronica, Informazione e Bioingegneria, Politecnico di Milano, 20133 Milano, Italy; matteo2.zago@polimi.it (M.Z.); martina.delfino@mail.polimi.it (M.D.S.); manuela.galli@polimi.it (M.G.); 2 Dipartimento di Meccanica, Politecnico di Milano, 20133 Milano, Italy; marco.tarabini@polimi.it (M.T.); cristina.ferrario@polimi.it (C.F.); 3 Dipartimento di Scienze Biomediche per la Salute, Università degli Studi di Milano, 20133 Milano, Italy; filippo.bertozzi@unimi.it

**Keywords:** gait analysis, spatio-temporal parameters, wearable sensors, decision trees

## Abstract

A promising but still scarcely explored strategy for the estimation of gait parameters based on inertial sensors involves the adoption of machine learning techniques. However, existing approaches are reliable only for specific conditions, inertial measurements unit (IMU) placement on the body, protocols, or when combined with additional devices. In this paper, we tested an alternative gait-events estimation approach which is fully data-driven and does not rely on a priori models or assumptions. High-frequency (512 Hz) data from a commercial inertial unit were recorded during 500 steps performed by 40 healthy participants. Sensors’ readings were synchronized with a reference ground reaction force system to determine initial/terminal contacts. Then, we extracted a set of features from windowed data labeled according to the reference. Two gray-box approaches were evaluated: (1) classifiers (decision trees) returning the presence of a gait event in each time window and (2) a classifier discriminating between stance and swing phases. Both outputs were submitted to a deterministic algorithm correcting spurious clusters of predictions. The stance vs. swing approach estimated the stride time duration with an average error lower than 20 ms and confidence bounds between ±50 ms. These figures are suitable to detect clinically meaningful differences across different populations.

## 1. Introduction

Clinical gait analysis is routinely performed by medical operators to assess ambulatory functional limitations in people with musculoskeletal or cognitive impairments [1,2], as well as to evaluate an individual’s quality of life, morbidity and/or mortality [3]. Spatio-temporal gait parameters (i.e., gait speed, stride duration, step length, step width, etc.) provide an immediate picture of an individual’s gait profile [1]. They can be used to predict fall risk [4,5,6] and/or to quantify rehabilitation outcomes [1,2,7,8,9,10].

In the last decade, considerable effort was devoted to provide valid and practical alternatives to overcome the limitations of traditional laboratory testing, namely, expensive equipment and time-consuming setup [11,12]. A clear research and development trend appeared towards systems able to capture people’s motion without expensive equipment and with limited expert knowledge required [13]. In this landscape, inertial measurements units (IMUs) emerged as a promising family of devices to enable daily-life, affordable, unobtrusive diagnosis and rehabilitation of gait in a wide plethora of cohorts, ranging from neurological diseases to stroke patients [12,14,15,16,17].

Among the most appealing advantages of IMU-based gait evaluation in daily routine activities is the opportunity to capture walking adaptations in response to environmental changes or perturbations [16], thus, substantially enhancing the ecological validity of testing [18]. This poses severe challenges to the development of algorithms able to provide accurate measurements despite the natural gait variability [16].

Estimating spatio-temporal parameters from IMU data is not a trivial task due to inherent sensors noise and drift problems [11]. This partly explains why these systems (despite the recent flowering of commercial products [11]) have achieved moderate and sometimes inconsistent performances to date [17], that in turn limited their widespread use for pervasive healthcare [19]. Drifts, in particular, are susceptible to produce large deviations in the calculated results when a double-integration-based sensor fusion approach is adopted [11,20,21]. This approach heavily depends on raw data quality even when an error state Kalman filter is applied to correct sensors’ data [5,14]. Most studies achieved gait events detection by sorting peaks, valleys, and zero-crossing in the signals [22,23]. Other algorithms exploited a combination of continuous wavelet transform to detect initial/final contacts (heel-strike, toe-off) and the inverted (double) pendulum model to extract spatio-temporal parameters from sensors’ readings. Examples exist where the sensing device was placed on the pelvis, typically in correspondence of the L4–L5 vertebrae [24,25] (assumed to approximate body center of mass location [26]), or alternatively on the foot, exploiting information on the angular velocity of leg swing and size to obtain stride and step lengths [10]. Solutions were also proposed where the temporal detection of gait cycles was based on the norm of the angular velocity of the foot relative to an empirical threshold [27].

A promising but still scarcely explored strategy for the estimation of gait parameters is based on machine learning techniques. In this context, spatio-temporal gait parameters are predicted using a set of features extracted from the IMU signals [11]. Zhang and collaborators used support vector regression models to estimate fundamental gait parameters from an IMU-equipped insole [19]. Hannink et al. used deep convolutional neural networks to map stride-specific IMU data to the resulting stride length, training the model on a publicly available database of 101 geriatric patients [5]. Stride length was estimated with an error of 0.01 ± 5.37 cm [5]. They did not, however, focus on temporal parameters.

The achieved accuracy claimed by most of the existing researches is in principle potentially feasible to enable clinical comparisons in terms of temporal parameters [28]. In particular, stride duration was mostly determined with an error (typically measured against a reference system such as optical motion capture, force platforms or instrumented walkways) lower than 50–60 ms [11,29]. However, the main weakness of these approaches is that they are reliable only for particular subjects’ conditions [19], for specific IMU placement on the body, for few protocols [22], or when combined with additional devices [19]. In this paper, we tested an alternative gait-events estimation approach based on machine learning algorithms which is fully data-driven and does not rely on a priori models or assumptions.

Section 2 will describe the experimental design, the equipment involved, the processing steps, and the statistical computations performed. Results will be shown in Section 3 and separately discussed in Section 4, where limitations and possible practical implications of the study are also reported; Section 5 contains concluding remarks and perspectives.

## 2. Materials and Methods

### 2.1. Experimental Design and Participants

This study involved the simultaneous collection of data with a commercial IMU device and a force platform during walking. Ground reaction force data were used as a reference. Forty healthy participants (19 women, 21 men) aged between 22 and 55 years voluntarily took part in the experimental sessions; inclusion criteria were: (i) no diagnosed gait-related impairments and (ii) ability to walk comfortably at different speeds. All of the subjects were able to complete the test and coped with the provided instructions. Only anonymized signals were analyzed and no clinical nor personal information was requested.

### 2.2. Experimental Setup

**Figure 1:**
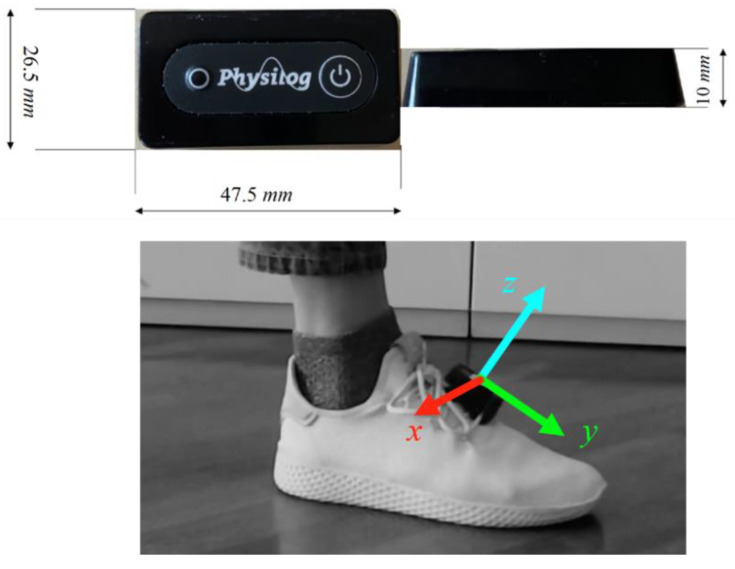
GaitUp Physilog size (**top**) and placement on the foot (**bottom**). Direction of the local right-handed reference frame axes is also reported.

A wearable IMU device (Physilog 5, GaitUp Ltd., Lausanne, Switzerland) was clipped to the participants’ right shoe in correspondence to the navicular bone (Figure 1). This IMU is a six-axes stand-alone unit integrating a three-axis accelerometer and a three-axis gyroscope. Device settings were set to a sampling frequency of 512 Hz, and a dynamic range of ±16 g for the accelerometer and 2000°/s for the gyroscope. The unit is sized 47.5 mm × 26.5 mm × 10 mm and weights 36 g. The sensors’ inertial right-handed coordinate system is oriented as displayed in Figure 1 (it is worth mentioning that GaitUp Ltd. also provides a commercial gait analysis solution, which has been validated and already used in several studies as described in [11]. As our goal was not to re-evaluate the GaitUp algorithms, only the raw data from the IMU development platform were processed in this study).

**Figure 2:**
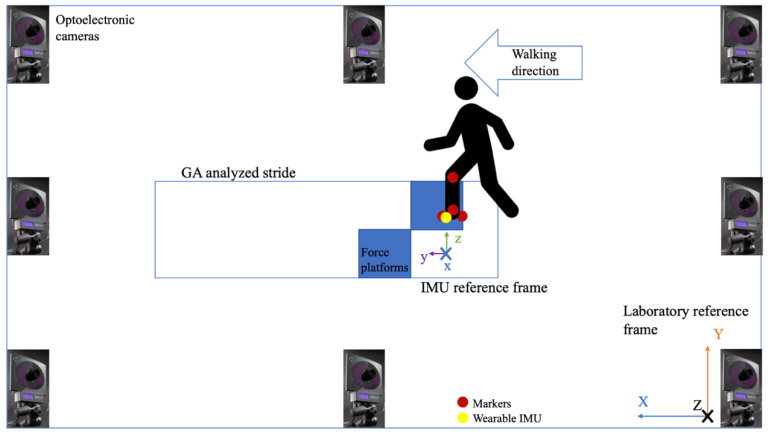
Experimental setup—tests were conducted on the middle laboratory lane with force platform embedded on the floor. Motion capture cameras are fixed on the wall in a standard gait analysis configuration.

A schematic representation of the laboratory setup is depicted in Figure 2. The laboratory was equipped with two 46.5 × 51.8 cm^2^ AMTI OR6-7 force platforms (Advanced Mechanical Technology, Inc., Watertown, MA, USA) sampling at 200 Hz, used to collect ground reaction forces (GRF) data. AMTI OR6-7 are strain gages-based force platforms designed for biomechanics applications. In the adopted configuration (10-V bridges excitation), full scale output in the medial and anteroposterior directions was 4450 N, while in the vertical direction it was 8900 N.

To provide a visual representation of the foot position during gait, the 3D position of four passive reflective markers was recorded with an optoelectronic motion capture system (SMART DX400, BTS Bioengineering, Milano, Italy) with a sampling frequency of 100 Hz. Markers were placed in correspondence to the lateral aspect of the foot at the fifth metatarsal head, on the heel, on the lateral malleolus and on the knee in correspondence to the lateral femoral epicondyle. Optical and ground reaction forces data are automatically synchronized. Global laboratory reference frame was oriented with the *x* axis horizontal and directed along the walking direction, the *y* axis pointing upwards, and the *z* axis mediolateral and pointing to the participant’s right (Figure 2).

### 2.3. Procedures

After starting the recording of both motion capture and IMU data, subjects were asked to hit a force platform three times with their right foot. This enabled to synchronize the two measurement systems (motion capture system and IMU) [30], as explained in the following paragraph.

Subsequently, all subjects performed a sequence of short straight-line level walking tests, from three to ten steps each, at self-selected comfortable speed, which were measured by means of the optical system.

### 2.4. Data Processing and Features Engineering

**Figure 3:**
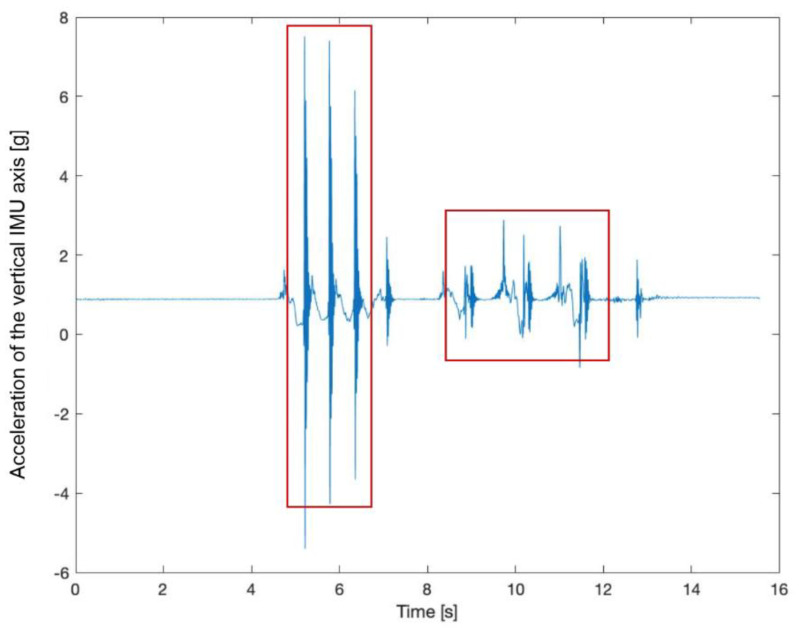
Sample vertical (*z*-axis) raw accelerometer readings during a test. The first three spikes correspond to the synchronization signal and the following data refer to gait events.

Data were processed by means of custom Matlab (v. 2019b, The Mathworks Inc., Natick, MA, USA) routines. GRF and inertial readings were time-aligned prior to each recording (maximum 30 s each) by determining the delay corresponding to the peak of the cross-correlation function among the vertical force and vertical acceleration signals at the beginning of the recording (Figure 3). The maximum synchronization error when dealing with cross correlation algorithms is typically lower than 1 sampling period [31]. In our case this is 10 ms, thus entailing a standard uncertainty lower than 3 ms. This value is trivial in comparison with the intrinsic variability of the observed phenomenon, as detailed later. Reference time events were obtained from foot–ground contact information, setting a binary GRF threshold of 10 N [32]. Stride time was computed as the time interval between two consecutive initial contacts of the right foot.

**Figure 4:**
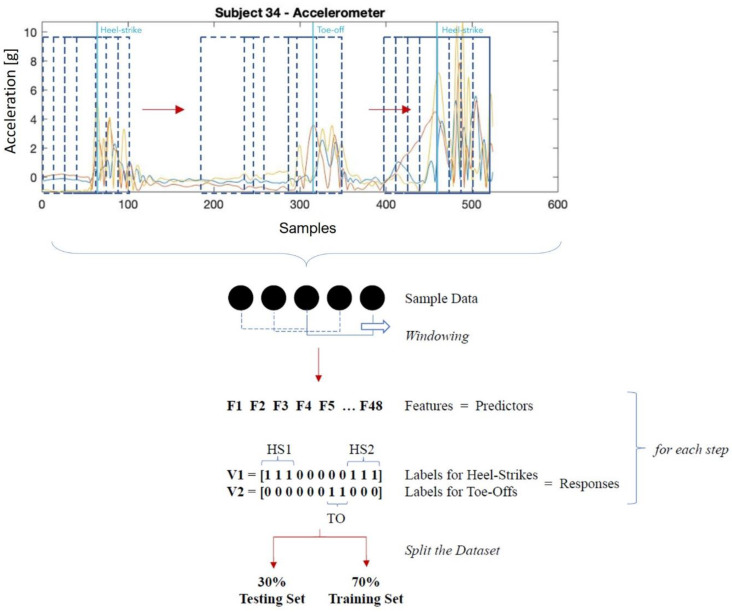
Data processing flow: sensors’ readings (in the top panel, sample acceleration signal) were windowed (dashed blue boxes represent the window moving across the signal); subsequently a set of features were obtained for each window, which was labelled according to the reference ground reaction force output. The whole set of collected strides (each one containing a collection of features) were randomly split into a training and a test set. HS: heel-strike, TO: toe-off.

The classification of gait phases and events followed a gray-box approach. First, sensor readings related to each step were trimmed in order to contain two consecutive heel strikes. The data stream was subsequently segmented in 64-sample (0.125 s) windows. A fixed-width, 64-sample moving window on the 6 IMU channels (acceleration and angular velocity) with step equal to 1 sample was used to compute 48 statistical features in the time and frequency domains, similarly to what previously done in [33]. In the time domain, the root mean square, variance, kurtosis, skewness, and correlation between each pair of accelerometer and gyroscope (angular velocity) axes were computed. In the frequency domain, the dominant frequency and the power at the dominant frequency were obtained (Table 1). Data processing flow is depicted in Figure 4.

### 2.5. Gait Events and Phases Classification

Three binary classifiers, supporting two alternative approaches, were implemented to identify:

- 1a: windows containing a heel strike vs. any other event.
- 1b: windows containing a toe off vs. any other event.
- 2: windows corresponding to stance vs. swing phases.

The results of gait event classifiers (1a) and (1b) were subsequently combined. Each window was labelled based on the reference output: in the case of gait events classifiers, label “1” was attributed to the windows containing a heel strike (1a) or a toe-off (1b), and label “0” elsewhere. In the case of classifier 2, label “0” was assigned to the swing phase and label “1” to the stance (ground contact) phase, evaluated at the time of each window’s first sample.

The whole sample of labelled features was split into a training (70% strides) and a test (30% strides) set. Of note, as the sequence of events is crucial to the problem of classifying gait phases, we randomly allocated strides (collection of consecutive observations), and no single observations.

The Matlab Machine Learning Toolbox identified decision trees as the most accurate binary classifiers relative to the concurrent application. Decision trees also allow for good classification speed and for features importance evaluation [33]. For these reasons, it was decided to base the following analysis on this classifier method.

**Figure 5:**
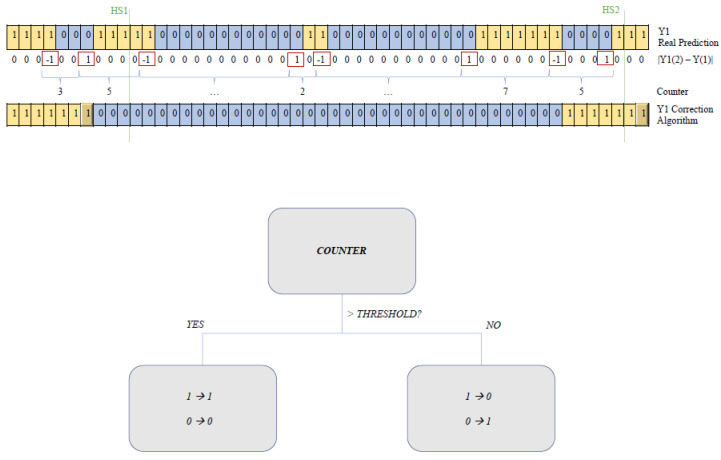
Correction algorithm: isolated short (counter < threshold) clusters were corrected according to the surroundings, as schematically reported in the bottom diagram.

The output stream returned by each classification approach was subsequently submitted to a “correction algorithm” aimed at detecting and removing isolated short clusters embedded in larger areas belonging to the opposed class. A cluster was considered “isolated” if it was shorter than half a window (i.e., 32 samples or 0.063 s), preceded and followed by a differently classified array of larger size (Figure 5). The correction algorithm works sequentially and “prefers” the current class. That is, if we are within a “stance” phase, short clusters labelled as “swing” are reversed into “stance”. The transitions between classes on the obtained output stream identified the correspondent gait event: for instance, the transition between the stance and the swing phase denoted a toe-off event.

### 2.6. Statistical Analysis

The error’s bias and random component in the identification of heel-strikes, toe-offs, stride, stance, and swing times were determined on the test set. To do so, the mean and standard deviation of the difference between the reference and the estimated value were computed, as well as the corresponding root-mean-square (RMS) error and 95% confidence intervals (95%CI). The standard error of the mean was computed for the whole test set (U) and for 10 strides (U_10_), the latter considering a common number of repetitions per subject in clinical gait applications [1].

Paired Students’ *t*-tests were performed between estimated and reference stride, stance and swing times, and associated with the corresponding Cohen’s d effect size (ES): values of d ≤ 0.5, 0.5 < d ≤ 0.8, and d > 0.8 were considered low, moderate, and large effects, respectively. The coefficient of determination (R^2^) was obtained between estimated and reference stride time, and between the related error and the corresponding gait speed. A statistical significance threshold of 0.05 was implemented throughout.

## 3. Results

Overall, participants performed 10–15 strides each, for a total of 500 recorded strides. Of them, 75 were discarded due to inconsistent or incomplete data. Therefore, the training set included 298 strides and the test set 127 strides. The global number of collected observations (64-sample windows) was 311,802.

**Figure 6:**
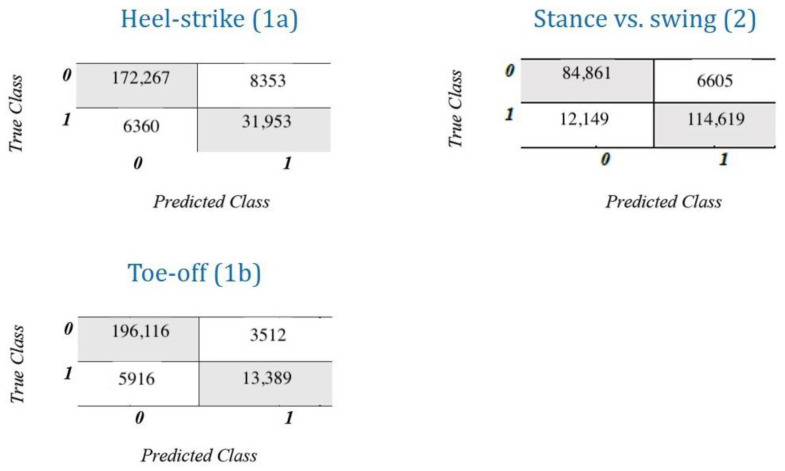
Classification performance of the three tested approaches. Left: heel-strike (**1a**) and toe-off (**1b**) events detection; Right: stance vs. swing moving windows classification (**2**).

Figure 6 and Table 2 report the output of the three binary classifiers: the gait events classifiers (1a and 1b) returned an accuracy of 91–93%, while the stance vs. swing classifier (2) reached 95.6% before being submitted to the correction algorithm. Some features were remarkably more predictive of the correct class. In particular, vertical acceleration played a substantial role in all the models, while angular velocity was less important to discriminate between stance and swing phases.

**Figure 7:**
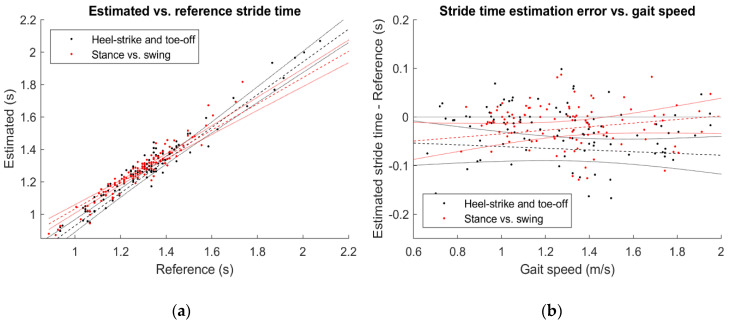
Regression plots comparing estimated and reference stride time (**a**) and the measurement error as a function of gait speed (**b**). Regression lines (dashed) and 95% confidence bounds (solid lines) were reported.

Globally, the stance vs. swing approach returned lower errors in determining all the considered parameters (Table 3). In particular, events identification returned an average error between −11 and 5 ms (95%CI, heel-strike) and between −13 and 50 ms (toe-off). Conversely, the approach involving methods 1a and 1b returned larger uncertainty values, reaching 35 ms (heel-strike) and 74 ms (toe-off) when considered over 10 strides. Gait phases estimation were therefore significantly different with small-to-medium effect sizes for the approach 1a–1b, while the stance vs. swing method showed no statistical differences for stance and swing phases duration (*p* = 0.098 and *p* = 0.782, low effect); the same approach underestimated the stride time by (*p* = 0.004, 95%CI between −37 and −7 ms) with a low effect size. However, as displayed in Figure 7a, the coefficients of determination were 0.796 and 0.808 for the gait-events classifiers (1a and 1b) and stance vs. swing (2) approaches, respectively (*p* < 0.001 for both).

In the best case (stance vs. swing approach), we found a single totally erroneous heel-strike (2% of the test set, difference from the reference of about 0.7 s) and seven toe-off events whose error was higher than 0.2 s (6% of the test set). There was not a significant correlation between the estimation error and the walking speed (Figure 7b), being R2 equal to 0.004 (*p* = 0.482, approach 1a and 1b) and 0.021 (*p* = 0.135, approach 2).

## 4. Discussion

By exploiting uncorrected, high-frequency acceleration and angular velocity data readings combined with a grey-box machine-learning approach, in this paper, we showed that it was possible to estimate gait events (heel-strikes and toe-off) with an average error lower than 20 ms and confidence bounds between ±50 ms. This led to determine stride time with a root-mean-squared error of about 80 ms.

### 4.1. A Data-Driven Approach

The discrimination of stance vs. swing phases (approach 2) outperformed the first approach (1a and 1b), intended to detect time windows where the initial/final contacts occur. This was partly expected, as gait events per se are distinct instants in time: a moving window, even if relatively short (64 samples at 512 Hz equals 125 ms) turned out to be prone to systematically anticipate (heel-strike, negative bounds of 95% CI) or delay (toe-off, positive bounds of 95%CI) the events detection. This inevitably led to significant systematic differences between the estimated temporal gait parameters (low-to-moderate effect sizes) and the reference system (force platform).

Conversely, the stance vs. swing approach almost halved the measurement error. Stride time resulted still significantly lower than that detected by the reference system, but with a low effect size and a reduced uncertainty (U_10_ = 25 ms). The RMSE of about 80 ms were slightly higher than a more sophisticated experimental setup based on an instrumented insole [19]. Confidence interval was limited to 29 ms and between the limits of agreement obtained by Yeo and colleagues [29] (60–100 ms), who also reported a very similar error of 20 ms. The correlation coefficient with the reference measure was r = 0.89, substantially in line with [11] and slightly lower than in Zhou and collaborators [16] (r = 0.95). Swing and stance time confidence intervals (−29, 38 ms and −50, 5 ms) were comparable with those provided by Godfrey and collaborators, who obtained (−35, 49 ms and −39, 49 ms) with a pelvis-mounted IMU and event detection based on Gaussian continuous wavelet transform [25].

Notably, these outcomes were purely data-driven. In other words, they were obtained without a priori assumptions, neither concerning subjects’ anthropometrics or speed nor regarding raw signal conditioning (i.e., filtering, sensors’ bias compensation), which was deliberately avoided to show the potential of the approach. Not relying on any deterministic model, the algorithms provided are potentially able to capture unconventional gait patterns, for instance with long stance phases and shorter steps than normal, as in the case of Parkinson’s disease [14,17,28].

Discrepancies from previous research performances should also be read in the light of the numerosity of the experimental cohort. Instead of gathering a large number of strides from a relatively reduced sample of participants, we decided to extend the survey to a wide (*n* = 40) range of subjects, higher than those observed in similar studies (e.g., five subjects in [11], 14 subjects in [19], and 30 subjects in [29]). This was done to enhance the generalizability of results, despite it could have introduced interindividual variability and reduced the estimation accuracy.

Besides their limited computational cost, an advantage of the regression trees used as the main classification algorithm is the opportunity to easily examine features’ relative contribution. Vertical acceleration [23] and anteroposterior angular velocity were the most revealing among a reduced set of discriminant statistical features. In that, the grade of variability of a signal throughout a time window (accounted for by the signal’s variance) was probably key to capture quick variations due to initial contact and/or toe-off. Additionally, limiting the number of relevant features adds to the feasibility of a real-time implementation. This reduces the computational burden often associated with end-to-end complex machine learning models (as deep convolutional neural networks [19]) or double integration approach (exploiting zero-velocity update technique), which requires sensors fusion algorithms [11]. Lastly, the proposed correction algorithm is simply enabling a sort of data cleaning prior to the determination of gait events, and its execution requires very simple and inexpensive operations.

### 4.2. Effect of Measurement Uncertainty in the Real Instrument Usage Context

Normative data in healthy adults report an interindividual variability (standard deviation) of stride time of about 80–120 ms [34]. This parameter tends to increase with age [34,35,36] and when a perturbation (physical, pathological, or cognitive as dual-task) arises, reaching values up to 180 ms [17,37]. Other investigations on orthopaedic patients reported a variability of 90 ms in the stride times measured in a single participant at self-selected speed across over ten gait cycles [8]. In this context, the obtained U_10_ value of 25 ms appears tolerable to properly characterize temporal gait parameters, even when the goal is detecting mean differences across populations: as a reference, Beauchet et al. reported between-sexes differences in stride time of about 50 ms [34], while Hollmann and collaborators denoted an increase in stride time higher than 60 ms between 70–74 years and 85+ years old women [35]. Significant differences of about 20–30 ms were also found between healthy controls and Down Syndrome patients at lower walking speed [38].

### 4.3. Limitations

The purpose of this study was to show the feasibility of classifying gait phases and determining the related events during walking in healthy adults. While we claimed that this method could be easily extended to other forms of locomotion like running or hopping, the proposed approach could not necessarily directly apply to patients with locomotor impairments. The collection of new training data in these conditions would be probably required. Likewise, in order to correctly capture the specific walking variability of pathological or pediatric populations it is advised that additional training data would be collected and combined. The real-time implementation of the algorithm was beyond the scope of this paper, and will be addressed in upcoming research.

A second limitation is that the original dataset was randomly split into a training and test set, without explicitly separate subjects. This was done to ensure the highest generalizability of the training set, but it also means that in principle, steps from the same participants could have be assigned to both splits.

The sampling frequency of the reference laboratory equipment (force platforms) was 200 Hz. This brought in an inherent uncertainty of 5 ms/2·$\sqrt{3}$ = 1.4 ms. In this sense, referring to instrumented walkways (e.g., Microgate’s Optogait [39]) the higher time resolution (up to 1 ms) would lead to a minor reduction in the overall measurement uncertainty. However, previous investigations relied on optical systems with a sampling frequency of 100 Hz to perform the same comparison [16]. Moreover, as previously discussed, differences in gait temporal parameters of this magnitude (<10 ms) are not clinically meaningful.

Lastly, we did not face the issue of multiple units’ synchronization, that, in principle, could be an additional source of uncertainty [40].

## 5. Conclusions

IMU-based solutions for the assessment of the gait function in real-world settings are continuously improved to provide personalized and pervasive healthcare [16]. In this study, we proposed a novel data-driven approach for the determination of temporal gait parameters based on inertial sensors and simple machine learning algorithms. Measurement errors were comparable to existing IMU-based methods, still, the proposed approach did not rely on any particular raw data processing constraint, and it was robust to inter-subject variability, thus, making it unnecessary to collect patient- or condition-specific training data [19]. Further, the proposed approach opens to further advancements on this path, which offers reduced computational burden and the potential to detect gait phases even when unconventional ambulatory modes are evaluated outside from restricted laboratory settings.

This study also reinforces the use of a movement analysis laboratory as a required reference when testing the measurement uncertainty of new devices. In the current market situation, in which we are witnessing the rapid spread of different systems for measuring a wealth of parameters related to human movement, it is necessary to adopt methods aimed at rigorously verifying the quality of the data produced.

## Figures and Tables

**Table 1:** Statistical and frequency-domain features.

Signal	Time Domain	Frequency Domain
Acceleration (3 channels)	Root mean squared	Dominant frequency
	Variance	Power at dominant frequency
	Kurtosis	
	Skewness	
	Linear Correlation (*x-y*, *x-z*, *y-z*)	
Angular velocity (3 channels)	Root mean squared	Dominant frequency
	Variance	Power at dominant frequency
	Kurtosis	
	Skewness	
	Linear Correlation (*x-y*, *x-z*, *y-z*)	

**Table 2:** Classifiers (decision trees) technical figures. The reported accuracy refers to the classification outcome, not to the final gait event estimation result. The outputs of Methods 1a and 1b were subsequently combined.

Item	Heel-Strike vs. Other (1a)	Toe-Off vs. Other (1b)	Stance vs. Swing (2)
Prediction accuracy	93.3%	91.4%	95.6%
Observations	218,933	218,933	218,933
Misclassification cost	14,713	18,754	9428
Prediction speed	7 × 10^6^ observations/s	7 × 10^6^ observations/s	6.9 × 10^6^ observations/s
Training time	52.145 s	18.137 s	49.694 s
Size of training data	87 MB	85 MB	87 MB
Validation	Hold-out	Hold-out	Hold-out
Features whose importance was greater than 5%	Mediolateral mean ω Vertical acc. RMS	Vertical acc. Mean AP ω mean AP ω var	Vertical acc. RMS AP acc. Mean

Acc.: acceleration, AP: anteroposterior, ω: angular velocity, RMS: root-mean-square; var: variance.

**Table 3:** Descriptive statistics (in ms) of the heel-strike and toe-off identification, as well as swing/stance/stride time estimation, with respect to the reference system (force platform).

Method	Mean	SD	RMSE	U	U_10_	95%CI	*p*	ES
*Heel-strike identification*								
Heel-strike and toe-off (1a, 1b)	−20	111	113	7	35	−33, −6	-	-
Stance vs. swing (2)	−3	59	59	4	19	−11, 5	-	-
*Toe-off identification*								
Heel-strike and toe-off (1a, 1b)	95	233	251	21	74	54, 136	-	-
Stance vs. swing (2)	19	165	166	15	52	−13, 50	-	-
*Stance phase estimation*								
Heel-strike and toe-off (1a, 1b)	−113	214	241	19	68	−150, −75	<0.001	0.514
Stance vs. swing (2)	−26	168	169	16	53	−50, 5	0.098	0.158
*Swing phase estimation*								
Heel-strike and toe-off (1a, 1b)	39	221	224	20	70	0, 78	0.048	0.205
Stance vs. swing (2)	5	179	178	17	56	−29, 38	0.782	0.034
*Stride time*								
Heel-strike and toe-off (1a, 1b)	−74	140	158	12	44	−98, −49	<0.001	0.258
Stance vs. swing (2)	−22	79	81	7	25	−36, −7	0.004	0.122

ES: Cohen’s d effect size; RMSE: root mean square error; U: standard error of the mean; U_10_: standard error of the mean on 10 strides; p: paired *t*-tests.

## Data Availability

The data presented in this study are available on request from the corresponding author.
